# Ozonated Olive Oil Dressing for Pediatric Hypospadias Repair: A Prospective Randomized Clinical Trial

**DOI:** 10.3390/children12050549

**Published:** 2025-04-24

**Authors:** Vincenzo Coppola, Maria Escolino, Fulvia Del Conte, Claudia Di Mento, Francesca Carraturo, Giovanni Esposito, Francesco Tedesco, Roberta Guglielmini, Ciro Esposito

**Affiliations:** 1 Pediatric Surgery Unit, University of Naples Federico II, 80131 Naples, Italy; maria.escolino@unina.it (M.E.); fulviadelconte@gmail.com (F.D.C.); claudia.dimento@unina.it (C.D.M.); francescacarraturo00@gmail.com (F.C.); frances.tedesco94@gmail.com (F.T.); dottrobertaguglielmini@gmail.com (R.G.); ciroespo@unina.it (C.E.); 2 CEINGE Advanced Biotechnology Center, 80131 Naples, Italy; repartochirped.fed2@gmail.com

**Keywords:** hypospadias, dressing, keyword, ozonide-based spray, wound, complications, children

## Abstract

Many ozone-based products that promote the healing process of wounds have been released in recent years. In this study, we evaluate a new spray dressing preparation based on stable ozonides with Vitamin E Acetate in children operated for distal hypospadias. **Methods**: We included all patients with distal hypospadias, who underwent Tubularized Incised Plate Urethroplasty (TIPU) over a 12-month period. The patients were randomized in two groups according to the type of medication: ozonide spray with Vitamin E Acetate (G1); hyaluronic acid cream (G2). After discharge, parents changed the dressing twice a day for 2–3 weeks postoperatively. The patients were evaluated at 7, 14, 21, 30, 60, and 180 postoperative days and thereafter annually. At the end of the treatment, we submitted a satisfaction questionnaire to parents. **Results**: Eighty-six patients (median age 18 months) were included. The wound healing was significantly faster in G1 compared with G2 (*p* = 0.001). No adverse skin reactions occurred in either group. Foreskin dehiscence and re-operation rates were lower in G1. Postoperative foreskin retractability was better in G1, with a significantly higher incidence of secondary phimosis in G2. The median treatment costs were significantly lower in G1 compared with G2 (*p* = 0.001). Group 1 found the spray dressing easy to use, improving patient management and adherence. **Conclusions**: The new preparation of ozonide-based product adopted promoted faster wound healing compared to conventional dressing. Furthermore, this spray preparation is easy to apply, economical, and simpler to preserve. This is better for parents who do not have to touch the wound to apply the product.

## 1. Introduction

Hypospadias is a congenital condition where the urethral opening is abnormally located on the underside of the penis due to incomplete closure of the urethral folds during fetal development. The severity of hypospadias can vary, with some cases involving a mild displacement and others more significant deformities [1], as may the choice of the most suitable surgical technique [2].

Treatment involves surgical correction to restore normal urinary and reproductive function, often performed between 6 and 18 months of age [3]. The tubularized incised plate urethroplasty (TIPU), described by Snodgrass, is among the most commonly used techniques [2]. Success depends on various factors including surgical staging, antibiotics, catheters, and materials like sutures and dressings [4]. Despite advancements, postoperative complications remain a challenge. Hypospadias still represents one of the most debated problems, not only among pediatric surgeons [5]. The risk of failure in hypospadias is quite high and depends on many factors. Among these, the need for a lower rate of postoperative complications and the possibility of using a protective barrier between the urethroplasty and the skin stand out [6]. This shows the importance of correct post-operative management and dressings [7]. An effective hypospadias dressing should reduce edema, prevent hematomas and infections, and serve as a protective barrier [8]. However, there is no consensus on the best dressing method. Medication based on hyaluronic acid, cyanoacrylate (CA), and others have been proposed in recent years [9].

Nevertheless, there is no evidence in the current literature of the best method for postoperative dressing following hypospadias repair [10]. An undervalued aspect is that the dressing should be simple to apply and to remove, as well as non-adherent to the incision [11,12].

In recent years, the use of ozone-based dressings for the treatment of surgical wounds has become very popular.

Ozone regulates key metabolic pathways by promoting oxidation-reduction reactions and activating enzyme functions, stimulates the endogenous defense system and, by regulating gene transcription, promotes damage repair and restoration of normal physiology. It also supplies oxygen to the tissues and acts as an anti-inflammatory and microbicide [13]. In addition, ozone products enriched with additives are coming onto the market, which have the function of enhancing the anti-inflammatory effect of the product. Among these, vitamin E acetate has particular interest. Studies have been carried out on the effectiveness of vitamin E acetate as an anti-inflammatory agent [14]. In pediatric urology, in fact, it is already used in place of corticosteroids to treat lichen in patients with phimosis, with satisfactory results [15].

Most oil-based products are available in gel formulations, which are difficult to manage. The purpose of our study is to evaluate the use of a new ozonide product with vitamin E acetate in a spray formulation that appears to be effective, easier to apply than other formulation, comfortable, with better parent compliance, and with less complications than simple conventional dressing.

## 2. Materials and Methods

We compared the use of two different types of dressings post-hypospadias surgery: a classic one by applying hyaluronic acid-based cream formulation (G2) and an ozonide-based dressing with Vitamin E Acetate in a spray formulation (G1). This prospective study was approved by the Committee of Medical Ethics and the institutional review boards of the authors’ institutions.

### 2.1. Enrolment

Only patients with distal hypospadias with a coronal urethral meatus, aged between 12 months and 3 years, who were candidates for Snodgrass urethroplasty (TIPU) and preputioplasty were enrolled. Patients who had previous hypospadias correction surgery were excluded from the study. Only patients with a coronal urethral meatus were included. Patients older than 3 years and patients who had previous hypospadias correction surgery were excluded from the study. Randomization was performed using opaque, sealed envelopes containing pre-labelled ballot papers for G1 or G2. Envelopes were drawn in sequential order immediately before surgery but only opened after completion of the surgical procedure. All patients were given prior information on the treatment, with information relating to the characteristics of both products used, and written informed consent was obtained from all parents.

### 2.2. Sample Size Calculation

The sample size was calculated assuming a margin of 15% for the absolute difference in rates. Considering a drop-out rate of 25%, 43 patients per arm would achieve 80% power to detect aim with a one side significance level (alpha) of 0.05.

### 2.3. Data Collection and Outcomes Measurement

**Figure 1:**
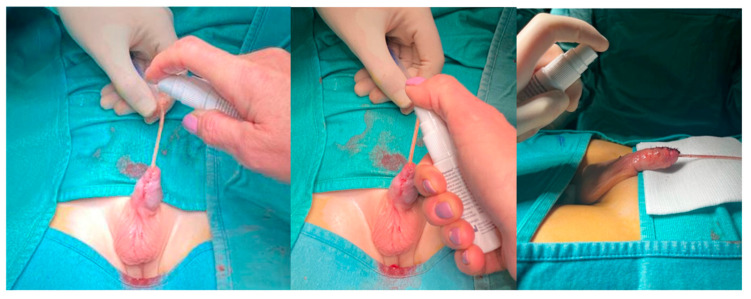
Spray formulation of Ozonide Oil gel for Hypospadias healing.

The first dressing was performed in the operating room after surgery. The dressing was then changed between the fifth and seventh day, coinciding with the removal of the bladder catheter. In the first few days after surgery, it was carefully explained how to apply the dressing in both groups. In the case of G1, after cleansing the wound, 2 sprays of the ozonated formulation (per 4 cm^2^ of wound) were applied from a vertical distance of approximately 5 cm directly above the surgical site, following the manufacturer’s recommendations for optimal diffusion and minimal pressure on tissues (Figure 1).

Parents were asked to touch the penis as little as possible, never touching the suture and exposing the skin by handling the dorsal area. Afterwards, gauze was placed again to protect and to promote a moist environment. This dressing was carried out morning and evening. In the case of G2, the parents placed an even layer of cream on the wound and perilesional skin, which was then covered with gauze. In no case were compression dressings performed. In both groups the dressing was carried out twice a day (morning and evening) for 2 weeks. The parents were encouraged to change the diaper frequently to avoid prolonged direct contact of the wound with the stools. Postoperative assessments were performed by four independent pediatric surgeons blinded to the treatment group. Parents were also instructed not to disclose the dressing type during follow-up visits to minimize potential bias. We carried out serial check-ups to evaluate the state of the wound (on days 7, 14, 30, 60, 180, and then annually). During each check-up, photographs were taken, and the following were assessed: wound status, healing, complications. The surgery was defined as complete and successful when and a normal appearance of the reconstructed foreskin without scars, irregularity, or asymmetry and a straight penile axis without curvature or torsion was revelated. The presence of a vertical aspect of the penile shaft with a wide meatus at the apical site of the glans was considered as a necessary condition to define the absence of complications. We adopted the SWAS score (Southampton Wound Assessment Scale) for the evaluation of post-discharge the surgical wounds (Table 1).

In the first few days’ post-surgery (day 1, 3, and 7), an assessment of the patient’s state of pain/irritability was carried out using a standardized scale for newborns: Face, Legs, Activity, Cry, Consolability (FLACC) Scale [16]. At the end of the follow-up (day 180), parents were then given a satisfaction questionnaire relating to the product used for the dressing, based on established questions to which they had to assign a score from 1 (not very satisfied) to 4 (extremely satisfied). The questions were related to the ease of use of the product, management, tolerance, ease of storage, and cost of the product.

### 2.4. Statistical Analysis

Statistical analysis was performed using SPSS software version 12.0 (SPSS, Inc., Chicago, IL, USA). Student’s *t*-test was used to compare continuous variables (age, duration of hospitalization, follow-up period, indwelling bladder catheter and wound healing time). A Chi-squared test or a Fisher’s exact test was used to compare the differences in post-operative complication, re-operation, and other wound characteristics. In all analyses, significance was defined as *p* < 0.05. Missing data due to withdrawal or incomplete follow-up were excluded from outcome analyses. An intention-to-treat (ITT) approach was adopted for all randomized patients.

The one patient from Group 1 who was lost to follow-up was included in the intention-to-treat analysis and considered as having completed the treatment without complications, in line with the pre-specified conservative assumption.

All statistical analyses were performed on an intention-to-treat basis.

### 2.5. Dressing Composition

The ozonated olive oil dressing (G1) used in this study was based on a stable ozonide formulation enriched with Vitamin E acetate, designed to have anti-inflammatory and antimicrobial properties. The preparation is alcohol-free and does not contain any synthetic preservatives. The hyaluronic acid cream (G2) contained sodium hyaluronate as the active ingredient in a water-based vehicle, with no alcohol or irritant preservatives. Both products were applied in their commercially available formulation without further modification.

## 3. Results

**Figure 2:**
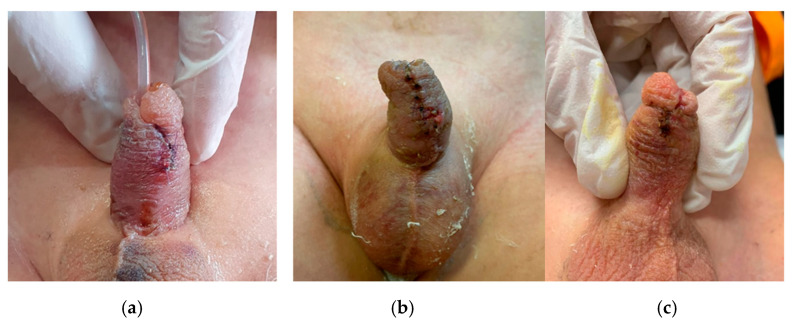
Result at day 3 (**a**), day 7 (**b**), and day 14 (**c**).

We included in the study eighty-six patients who were randomized in two groups: forty-three patients (treatment group, G1) treated with Ozonide spray, and forty-three patients (control group, G2) treated with normal dressing with hyaluronic acid cream. Age distribution, the proportion of hypospadias degree, hospital information, and patient’s baseline/demographics are summarized in Table 1. Only one patient in Group 1 was lost during follow-up due to withdrawal of informed consent. The mean wound healing time (in days) was significantly shorter in the G1 group than in the control group (17.2 vs. 31.3) (*p* < 0.05). Figure 2 illustrates the healing progression in a representative patient of the G1 group at day 3, 7, and 14. No adverse reactions to the product were reported in either study group. The mean follow-up time and mean hospitalization time were not statistically significant. The bladder catheter was removed between the 5th and 7th postoperative day in G2 and the 3rd and 6th day in G1. All patients were discharged the day following the catheter removal. Considering other wound characteristics within 30 days after surgery, and inflammation (7 vs. 18) and swelling (6 vs. 12) appeared in the study group in more than 50% fewer cases than in the control group (*p* < 0.05). The incidence of postoperative complications including infection, foreskin dehiscence, urethral meatus stenosis, and urethra-cutaneous fistula was evaluated. Postoperative complications were graded according to Clavien-Dindo grading system. In the G2 group, seven complications were reported, while in G1, there were two (*p* > 0.05). No infections were reported in G1, compared with G2, where there were two cases of infection treated with antibiotic therapy. In G1, reintervention was required in two cases (two circumcisions for foreskin dehiscence), whereas in G2, reintervention was required in five cases (one redo-TIPU, one urethro-cutaneous fistula closure, two circumcisions for foreskin dehiscence, and one redo-prepuzioplasthy for closure of a foreskin fistula). The re-operation rate was lower in G1 [(2/43) 4.7%] compared with G2 [(5/43) 11.6%] (Table 1). Other wound characteristics on day 7 were also analyzed (Table 2).

Assessment of pain and irritability according to the FLACC Scale of infants at days 1, 7, and 30 reported significantly better values for G1 than G2 (*p* < 0.05), with lower pain assessment as early as 7 days after surgery. After 6 months, a questionnaire evaluating the product used was administered, which reported better ease of application of the spray product than the cream formulation. In terms of ease of application, time required, reliability, and cost, the G1 group was clearly superior (*p* < 0.05). The results for the FLACC scale and questionnaires are shown in Table 3.

## 4. Discussion

This prospective randomized study showed that the use of an ozonide-based spray dressing after distal hypospadias repair promoted faster wound healing with higher parental satisfaction compared to a standard hyaluronic acid cream. The importance of post-operative management of patients who have undergone hypospadias surgery is widely recognized. The surgical dressing likely represents the greatest variable and source of the controversy in postoperative care [7]. A wide variety of dressing options are available: compressive, non-compressive, and no dressing have been described for hypospadias repair, although no clear consensus regarding dressing has been established [17]. Nonetheless, some interviews carried out by surgeons from the pediatric urology society revealed that it is very common practice to apply a dressing post-operatively. The same study reported that a non-compressive dressing was favored by approximately half of respondents regardless of hypospadias severity, though a significant minority of respondents preferred a compressive dressing, especially for more severe hypospadias repairs [4].

The presence of a diaper likely increases the risk of wound contamination by stools, leading to potential infection. Therefore, it is advisable to incorporate the application of a dressing following hypospadias surgery. In our study, the group treated with the ozonide spray dressing showed faster wound healing within the first postoperative week compared to the control group. This confirm that a dressing also acts as a valuable mechanical barrier against tissue contamination and helps to reduce post-operative edema (Figure 2).

As outlined in the existing literature, an optimal dressing should possess physical attributes such as elasticity, durability, and flexibility, while exerting effective pressure on the wound [12]. Additionally, the dressing should induce minimal adverse reactions upon contact with tissues and should facilitate easy, painless removal [12,18,19]. Several types of dressing materials have been proposed, including petrolatum-based gauze, silastic foam, elastic bandage, finger glove, Tegaderm™, Opsite^®^, Cavi-Care^®^, Granuflex^®^, Dermolite™, and Coban™ bandage [7,11]. These can then be enriched with creams or medicines to aid the healing process. Product based on Chamomile and Lidocaine hydrochloride gel, Aloe vera, or autologous platelet gel might decrease pain and edema and reduce inflammation and fibrosis. In addition, tubular finger oxygen-enriched oil inside-coated devices were produced, proving to be very effective in reducing the risk of complications [20,21]. In recent years, much attention has been focused on the use of ozone, showing its usefulness in the treatment of wounds with significant improvements in healing outcomes and healing time when compared with the standard care [22,23,24,25]. However, spray-based formulations have never been described before.

Among the advantages of the use of an ozone-based products observed in the literature, the reduction in the incidence of post-operative complications, such as phimosis and preputial adhesions, seems relevant [26]. The decreased incidence of foreskin dehiscence and reoperations observed in G1 may be attributed to both the biological properties of the ozonated product and its spray formulation. Unlike ointments, the spray allows for non-contact application, potentially reducing local trauma, wound contamination, and variability in parental administration. These elements may explain the lower postoperative complication rate in G1 (4.6%) compared to G2 (16.3%). This difference, although not statistically significant, is clinically relevant and supports the potential benefit of using a spray formulation for home-based wound care in pediatric urology, which allows you to handle the wound as little as possible and considerably reduce the risk of infections and complications in the first weeks of treatment.

It is also important to highlight that catheter removal timing differed between the two groups. This difference may have contributed to variation in outcomes, since earlier catheter removal can reduce the risk of infection and improve tissue perfusion. However, in this study, catheter removal timing was tailored to individual wound healing status, and no early removal led to complications in either group.

Less wound handling could explain the reduction in pain and irritability in children assessed in the first few weeks with the FLACC scale. In fact, many surgeons who do not use dressings in the postoperative period believe that wound handling should be minimized to reduce the risk of complications [18]. This finding could be encouraging to remedy this problem and have proper management in the postoperative period. Furthermore, another disadvantage when using ozonide oil is that many formulations must be stored in the fridge at temperatures lower than 15 degrees, and with each application they must be used after 5–10 min at ambient temperature so that it becomes fluid. The Ozonide spray formulation, however, can be stored at room temperature and is immediately ready for use. We also believe that enrichment of the product with Vitamin E acetate is an important element for increased anti-inflammatory efficacy [15], as demonstrated by the presence of a less red wound in the early post-operative days.

Regarding costs, the satisfaction questionnaire reported a clear preference in the use of ozonide when compared to hyaluronic acid. This is also because in Group 2, many more vials are needed to carry out the treatment. Furthermore, among the various ozone-based formulations available on the market, ozonide is the one that is much less expensive, with an average cost three times lower than the others.

The following study has some limitations. First, there are some possible confounding factors related to differences regarding the type of surgical technique, the anatomical characteristics of the child, the different postoperative management, especially regarding the dressings performed by the parents at home, and the rate of postoperative complications associated with hypospadias repair. To overcome this, we have tried to standardize and inform parents as much as possible about the dressing methods (thorough cleansing, number of sprays, dressings carried out, constant changing of diaper). Another limitation is related to the patient sample and follow-up. To have more reliable results it would be necessary to have a larger series analyzed over a long period of time. The ideal would be to follow these patients until puberty.

Another relevant limitation is the potential variability in dressing application, as the ointment (G2) required direct contact with the wound, while the spray (G1) could be applied without touching the surgical area. Although all parents received standardized instructions and demonstrations on dressing care, the involvement of different individuals at home may have introduced inconsistencies. This limitation could have been reduced by having a single operator or trained medical team perform all postoperative dressing changes.

In conclusion, in our series, we adopted a new preparation of ozonide-based product that promoted faster wound healing compared to conventional dressing. Furthermore, this spray preparation is easy to apply, stable at room temperature, and can therefore be considered a good choice for parent, who, to carry out the medications, do not have to touch the wound with their hands to apply the product.

## Figures and Tables

**Table 1:** Patients baseline/Demographics and outcomes.

	G1 N = 43	G2 N = 43	Adjusted Treatment Effect (+/−)	*p* Value
Median patients age (months)	19 ± 4.5	18 ± 5.2	n/a	n/a
Coronal Hypospadias, n (%)	37/43 (86%)	35/43 (81%)	+5%	>0.05
Subcoronal Hypospadia, n (%)	6/43 (14%)	8/43 (19%)	−5%	>0.05
Hospital stays (days)	6 ± 0.6	6 ± 0.6	0	>0.05
Follow-up (months)	15.0 ± 3.9	17.0 ± 6.3	−15.2%	<0.05
Indwelling bladder catheter (days)	4.3 ± 0.3	6.3 ± 0.7	−4.6%	>0.05
Wound healing time, (days)	17.2 ± 5.1	31.3 ± 16.9	−32.8%	<0.05
Postoperative complications, n (%)	2/43 (4.6%)	7/43 (16.3%)	−12.3%	>0.05
Urethrocutaneous fistula, n (%)	0	2/43 (4.6%)	−4.6%	>0.05
Foreskin dehiscence, n (%)	2/43 (4.6%)	2/43 (4.6%)	0	>0.05
Meatal stenosis, n (%)	0	1/43 (2.3%)	−2.3%	>0.05
Wound infection, n (%)	0	2/43 (4.6%)	−4.6%	>0.05
Total re-operations, n (%)	2/43 (4.6%)	5/43 (11.6%)	−7%	>0.05
Adverse skin reaction, n (%)	0	0	n/a	n/a

**Table 2:** Other wound characteristics on day 7.

	No./Total No. (%) G1	No./Total No. (%) G2	Absolute Risk Difference (95% CI), %	Relative Risk (95% CI)	*p* Value
**Wound Healing Complication**					
Red and inflamed	7/47 (14.9)	18/47 (38.3)	−23.4% (−38.6 to −8.1)	0.39 (0.18 to 0.83)	<0.05
Swollen	6/47 (12.7)	12/47 (25.5)	−12.8 (−28.4 to 2.8)	0.50 (0.20 to 1.24)	>0.05
Fever > 38 °C	3/47 (6.4)	4/47 (8.5)	−2.1 (−13.3 to 9.1)	0.75 0.17 to 3.26)	>0.05
Fluid Leaking (not pus)	12/47 (25.5)	14/47 (29.8)	−4.3 (−21.1 to 12.5)	0.86 (0.44 to 1.69)	>0.05

**Table 3:** Results for the FLACC scale and evaluation form of dressing used.

	G1 n = 43	G2 n = 43	*p* (X2)
**Evaluation of the dressing used**			
**Easy of Application**			0.0006
Excellent	29 (67.5%)	13 (30.2%)	
Good	9 (21%)	9 (21%)	
Fair	5 (11.6%)	20 (46.5%)	
Poor	0 (0%)	1 (2.3%)	
**Time Spent**			0.0087
Excellent	31 (72%)	19 (44.2%)	
Good	10 (23.2%)	12 (28%)	
Fair	2 (4.6%)	7 (16.2%)	
Poor	0	5 (11.6%)	
**Status and Cleanliness**			0.56
Excellent	35 (81.4%)	37 (86%)	
Good	8 (18.6%)	5 (11.6%)	
Fair	0	1 (2.3%)	
Poor	0	0	
**Comfort and Safety**			0.66
Excellent	18 (41.8%)	20 (46.5%)	
Good	15 (34.9%)	11 (25.6%)	
Fair	10 (23.2%)	12 (28%)	
Poor	0	0	
**Reliability**			0.0006
Excellent	28 (65.1%)	12 (28%)	
Good	11 (25.6%)	18 (41.9%)	
Fair	5 (11.6%)	12 (27.9%)	
Poor	0	2 (4.6%)	
**Cost**			0.0001
Excellent	33 (76.7%)	3 (6.9%)	
Good	9 (20.9%)	8 (18.6%)	
Fair	1 (2.3%)	13 (30.2%)	
Poor	0	19 (44.1%)	
**Face, Legs, Activity, Cry, Consolability (FLACC) Scale**			**P (t)**
**FLACC Scale at t.1**	8 (6–10)	6–7 (4–9)	0.0001
0: relaxed and comfortable			
1–3: mild discomfort			
4–6: moderate pain			
7–10: severe discomfort of pain or both			
**FLACC Scale at t.3**	4 (3–5)	5–6 (4–7)	0.01
**FLACC Scale at t.7**	1 (0–2)	3 (1–5)	0.0001

## Data Availability

The data presented in this study are available on request from the corresponding author.

## Abbreviations

The following abbreviations are used in this manuscript:

TIPU	Tubularized Incised Plate Urethroplasty
SWAS	Southampton Wound Assessment Scale
FLACC	Face, Legs, Activity, Cry, Consolability Scale
